# Role of Vacancy Defects in Reducing the Responsivity of AlGaN Schottky Barrier Ultraviolet Detectors

**DOI:** 10.3390/nano12183148

**Published:** 2022-09-11

**Authors:** Yujie Huang, Jing Yang, Degang Zhao, Yuheng Zhang, Zongshun Liu, Feng Liang, Ping Chen

**Affiliations:** 1 State Key Laboratory of Integrated Optoelectronics, Institute of Semiconductors, Chinese Academy of Sciences, Beijing 100083, China; 2 College of Materials Science and Optoelectronics Technology, University of Chinese Academy of Sciences, Beijing 100049, China; 3 Center of Materials Science and Optoelectronics Engineering, University of Chinese Academy of Sciences, Beijing 100049, China

**Keywords:** Ga vacancy, responsivity, AlGaN Schottky detector, MOCVD

## Abstract

The spectral response properties of AlGaN Schottky barrier detectors with different Al content were investigated. It was found that the responsivity of AlGaN detectors decreases with increase in Al content in AlGaN. It was found that neither dislocation density nor the concentration of carbon and oxygen impurities made any remarkable difference in these AlGaN devices. However, the positron annihilation experiments showed that the concentration of Al or Ga vacancy defects (more likely Ga vacancy defects) in AlGaN active layers increased with the increase in Al content. It is assumed that the Al or Ga vacancy defects play a negative role in a detector’s performance, which increases the recombination of photogenerated carriers and reduces the detector responsivity. It is necessary to control the concentration of vacancy defects for the high performance AlGaN detectors.

## 1. Introduction

AlGaN-based optoelectronic devices have many applications in laser diodes, HEMT, photodetectors and other devices [1,2,3]. By using AlGaN materials with high enough Al composition, visible-blind and solar-blind AlGaN-based detectors can be achieved and be employed to further increase the sensitivity and accuracy of UV detectors [4]. Researchers have successively developed p-n junction [5], p-i-n [6,7,8], metal/semi-metal [9] (MSM) and Schottky barrier [10,11] structures GaN-based UV detectors. Among these structures, the Schottky barrier detector is relatively simple and has the advantages of short response time and high quantum efficiency [12]. Therefore, Schottky barrier detectors are favored for UV detection, but the low responsivity limits the practical application of AlGaN UV detector, and responsivity is closely related to material quality. Researchers have studied the effect of dislocation density and carbon impurity concentration on the responsivity of GaN detectors [13,14,15]. In addition, the effect of vacancy on Al_x_Ga_1-x_N (0.08 < x < 0.3) devices with high Al content has been studied before [16]. However, the factors affecting the responsivity of Al_x_Ga_1-x_N (x < 0.07) UV detector with low Al content have not been clearly discussed. Therefore, this paper assumes that a series of AlGaN detectors with different low Al contents are prepared to study the factors affecting their responsivity. This will provide technical support for the preparation of high performance AlGaN near-ultraviolet (NUV) detector.

In this paper, the properties of the spectral response of AlGaN Schottky barrier UV detectors with different Al content AlGaN are investigated, where the Al content does not exceed 7%. It is found that the responsivity of AlGaN detectors decreases with the increase in Al content. A further study shows that there are more Al or Ga vacancy defects in the AlGaN materials with higher Al content. It is believed that the Al or Ga vacancy defects play a negative role in detector’s performance. The concentration of point defects in a material depends on its growth environment. Thus, finding the appropriate experimental conditions to reduce the concentration of vacancy defects is a direction of future research. Therefore, it is very important to improve the high responsivity AlGaN UV detectors with low Al content.

## 2. Experimental

**Figure 1:**
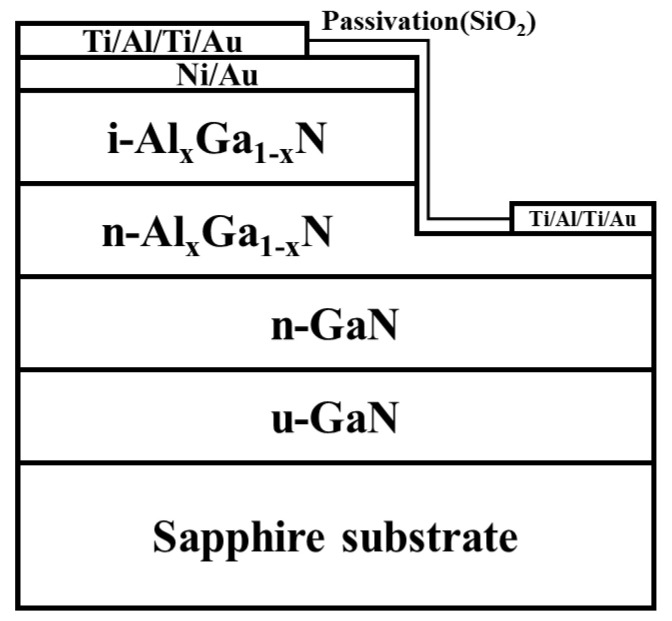
The cross-sectional scheme of the fabricated Al_x_Ga_1−x_N Schottky photodetector.

Five metal-Al_x_Ga_1-x_N Schottky barrier detectors, named as T0, T1, T2, T3 and T4, were grown by metal–organic chemical vapor deposition (MOCVD) on c-plane sapphire substrates. Their schematic diagram is shown in the Figure 1. Trimethylaluminium (TMAl), Trimethylgallium (TMGa), and ammonia (NH_3_) were used as precursors in the epitaxial growth process, respectively. Samples were prepared as follows. First, in order to reduce the lattice mismatch, an unintentionally doped GaN buffer layer was grown on the sapphire substrate. Next, a 1.7 μm n-GaN layer, a 500 nm n-Al_x_Ga_1-x_N layer and a 250 nm unintentionally doped i-Al_x_Ga_1-x_N layer were grown sequentially. The n-GaN layer and the n-Al_x_Ga_1-x_N layer were doped with Si. The five Schottky detectors had AlGaN active layers with different Al composition. The different Al content was obtained by adjusting the TMAl flow rate. The TMAl flux employed during the growth of Al_x_Ga_1-x_N layers was 0 μmol/min, 2 μmol/min, 3 μmol/min, 4.5 μmol/min, and 6 μmol/min for samples T0–T4, respectively. After the epitaxial growth, the detectors were fabricated through processing such as photolithography, etching, and coating to make Schottky photodetector devices. To ensure the Schottky contact and reduce the loss of light on the metal, most of the surface was covered by a thin metal layer of 10/10 nm Ni/Au. The transparency value of the 10/10 nm Ni/Au is about 60–75%. Above that, a metal layer of Ti/Al/Ti/Au which had a thickness of 15/150/50/150 nm was used to make electrical contact.

The in-plane ω-2θ and ω scan high resolution X-ray diffraction (HRXRD) measurements were performed by using a Rigaku SmartLab X-ray Diffractometer. The full width at half maximum (FWHM) at (002) and (102) reflection obtained by ω-scan rocking curves can help to determine the edge and screw dislocation densities in the material. The composition information of alloy materials was determined by ω-2θ scanning mode and GlobalFit software fitting. In addition, the impurity concentration distribution in the samples was measured by secondary ion mass spectroscopy (SIMS), and positron annihilation was used to measure the vacancy defects of samples. Moreover, photoluminescence (PL) results of the samples were measured with a 325 nm laser as the excitation source. Furthermore, in the spectral response test system a xenon lamp was used as the light source, followed by a monochromator. A calibrated Si detector was used to determine the accurate value of responsivity.

## 3. Results and Discussion

**Figure 2:**
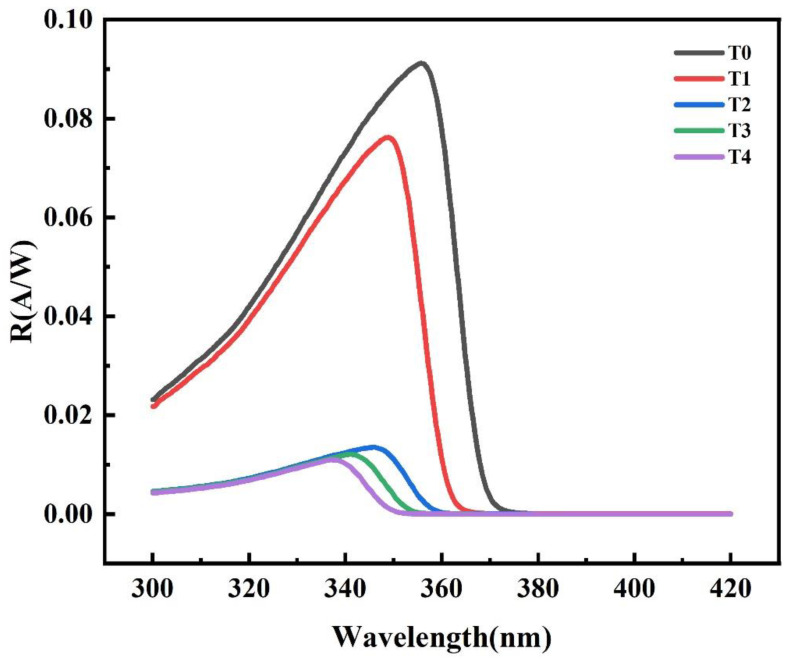
The responsivity versus wavelength for five samples T0–T4 under zero bias.

Table 1 shows that the Al content of T0–T4 samples obtained by ω-2θ HRXRD scanning mode and fitting with GlobalFit software. It should be emphasized that the Al content of T0–T4 samples increases as determined by this measurement method. Figure 2 shows the spectral response of five AlGaN Schottky junction detectors at zero bias voltage. Firstly, at the longer wavelength side of peak, there is a cut-off of responsivity at the band edge of samples. In addition, the cutoff wavelength of these five samples is different, corresponding to different Al components in AlGaN. Secondly, at the shorter wavelength side of the peak the absorption coefficient is larger, and the penetration depth of incident light is shorter. In this case the penetration depth of light will be shorter, and the surface recombination becomes stronger; thus the measured responsivity value will reduce with decreasing wavelength. Most importantly, it is noticed that the peak responsivity is measured as 0.091 A/W at 358 nm for T0, 0.076 A/W at 349 nm for T1, 0.014 A/W at 346 nm for T2, 0.012 A/W at 341 nm for T3, and 0.011 A/W at 337 nm for T4, which means that the peak responsivity decreases significantly with the increase in Al content in the AlGaN layer. 

In the next sections, we explain how we conducted a series of experiments to investigate the reason for the observed difference in the responsivity of the five detectors. An increase in dislocation defects or the concentration of carbon and oxygen impurity may lead to a decrease in the responsivity of Schottky detectors [17]. The dislocation defects may act as charge traps that can increase the recombination probability of photo-generated electron-hole pairs. In addition, the photo-generated carriers cannot be effectively collected because of carbon impurities-induced deep level centers [13]. The FWHMs of the ω-scan rocking curves measured by HRXRD are closely related to the edge and screw dislocation densities. The carbon and oxygen impurity concentration of the AlGaN layer measured by SIMS is also listed in Table 1. Note that the XRD FWHM values for samples T0–T4 are nearly the same, which are about 285 arc sec for the (002) and 305 arc sec for the (102) planes. However, the responsivity of samples T0–T4 has quite a distinct difference. This suggests that the different responsivity of these five detectors is not strongly dependent on the dislocation density. The SIMS result shows that the carbon and oxygen impurity concentrations of the T1–T4 samples are also very close to each other. However, the carbon and oxygen impurity concentration of sample T0 is slightly higher than that of T1–T4, which may be because a higher TMGa flux is used during the growth of sample T0. However, as shown in Figure 2, the responsivity of sample T0 is higher than that of samples T1–T4. This implies that a higher carbon and oxygen impurity concentration may only have a weak effect, or even no remarkable influence on the responsivity of the studied samples. In summary, it is found that neither dislocation density nor the carbon and oxygen impurity concentration have any essential influence on the different responsivity of the five samples in our experiments. Therefore, we postulate that point defects in the Al_x_Ga_1-x_N layers may affect the responsivity of the five samples.

**Figure 3:**
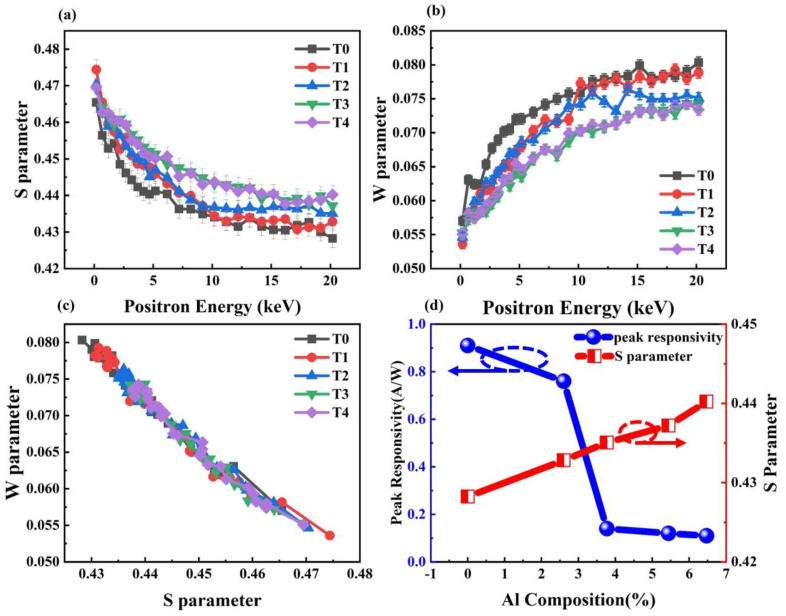
(**a**)The dependence of the low momentum parameter *S* on positron incident energy in the five samples. (**b**)The dependence of the high momentum parameter *W* on positron incident energy in the five samples. (**c**)The relationship between the low−momentum parameter *S* and the high−momentum parameter W of the five samples. (**d**) Peak responsivity and *S* parameter versus Al composition in AlGaN (The connected lines are used only for helping eyes).

Positron annihilation measurement is known to be a powerful tool for characterizing the concentration of point defects in materials [18]. Therefore, we carried out positron annihilation experiments to gain further insight into the possible effect of Al composition on point defects in Al_x_Ga_1-x_N. In these tests, the Doppler broadening spectra of the annihilation lines were recorded with a high-purity Ge detector. The broadening is conventionally described by low momentum parameter *S* and high momentum parameter *W* [18]. Figure 3a,b show the *S* parameter and *W* parameter as a function of positron incident energy for the five samples. It is observed that as the Al composition increases, the *S*-parameter value increases and the *W*-parameter value decreases. The certain vacancy defects in Al*_x_*Ga_1-*x*_N are mainly negatively charged [19]. When positrons are annihilated at metal vacancies, the value of parameter *S* will increase and that of parameter *W* will decrease, since a larger fraction of annihilation happens to low momentum electrons [20]. Therefore, the results in Figure 3a,b suggest that the increase in the certain vacancy defects concentration with the increase in Al content. Combined with the result shown in Figure 2 that the optical responsivity of the samples with higher Al content is lower, this suggests that there may be a correlation between the responsivity of detectors and the concentration of the certain vacancy defects. In order to further obtain the information about the point defects existing in the five samples, the relationship between the parameters *S* and *W* of these samples was investigated. In addition, the vacancy concentrations are estimated using the S and W parameters and listed in Table 1 [21]. As shown in Figure 3c, it is observed that the parameter *S* varies linearly with the parameter *W* in five samples. The slopes of the two curves are almost equal. This result indicates that only one type of point defect exists in these devices. Since this defect is a negative center and the material of the i-layer is unintentionally doped -Al_x_Ga_1−x_N, it is most likely to be a metal vacancy, that is, a Ga vacancy or an Al vacancy. According to research by Warnick [22] and Puzyrev [23], environmental conditions influence vacancies formation energies. Especially, the formation energy of Ga vacancy is slightly lower than that of Al vacancy, which means that Ga vacancy is easier to form in AlGaN material. Therefore, we think this defect is more likely Ga vacancy.

Figure 3d shows the relationship between responsivity and Al content and the relationship between S parameter and Al content. It can be seen that the higher Al content, the higher the concentration of Al or Ga vacancies. At the same time, the responsivity decreases with the increase in Al content. It is worth noting that their relationship between S parameter and responsivity is not linear. The responsivity of T0–T4 showed a downward trend on the whole, and the vacancy increased significantly. It suggests that the photo-generated holes can be trapped by the Ga vacancies. The concentration of movable photogenerated holes in the depletion region has a strong influence on the responsivity. The Al or Ga vacancies will trap photogenerated carriers or increase their recombination probability [24], leading to a serious reduction in responsivity. This indicates that Al or Ga vacancies may play an important role in the reduction in responsivity of AlGaN Schottky photodetectors. At the same time, the increase in Al or Ga vacancies may be caused by the increase in Al content during the epitaxial growth of AlGaN. We speculate that point defects are formed due to the deviation of the composition from the normal chemical ratio. With the increase in the TMAl flux, the reaction speed in the gas environment will increase. The Al or Ga atoms will have not enough time to migrate to the right crystalline sites, thus a Al_x_Ga_1-x_N crystal with stable ratio of chemical composition is difficult to form. Therefore, the concentration of Al or Ga vacancies will increase with increasing Al content.

**Figure 4:**
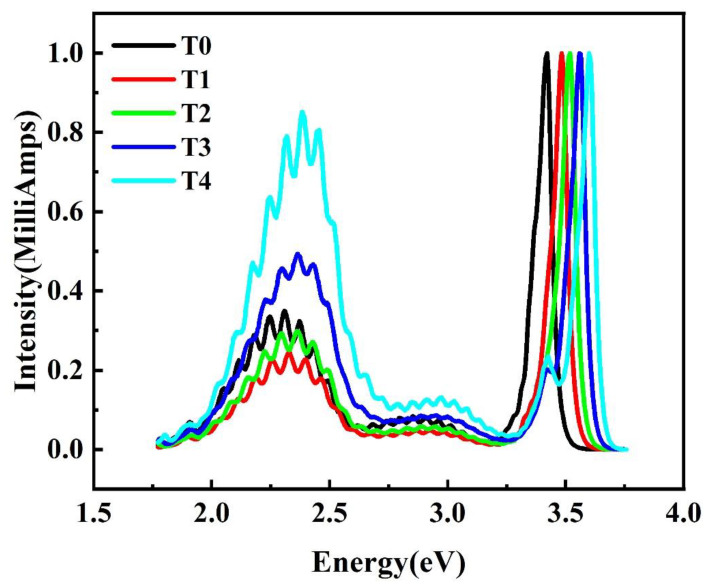
Normalized PL spectra at room temperature for five samples.

The room temperature photoluminescence (PL) spectra should be possible to provide important information of defects and their influence in AlGaN. The PL spectra of the five samples are measured and shown in Figure 4. The spectral intensity is normalized to the AlGaN intrinsic transition peak intensity near the band edge, providing strong evidence to support the point of view mentioned above. There is a shoulder peak located at 3.4 eV for samples T3 and T4. We assign it to the band edge luminescence peak of GaN. It is interesting to note that the intensity of a broad yellow luminescence (YL) band increases with the increase in Al content (for Al_x_Ga_1-x_N samples of x ≠ 0). The YL is strongly related to the deep level defects in the GaN and AlGaN materials, which will affect the device performance. It is known that the origin of YL is related to carbon impurities [25], dislocations [26] and vacancy defects [27]. Based on the result of SIMS and HRXRD, the dislocation density and the concentration of carbon impurity of samples T1~T4 is almost the same. (The carbon impurity concentration of T0 is a little higher, which may lead to a slightly higher intensity of YL). In addition, according to the result of positron annihilation the density of vacancy defects increases remarkably with increasing Al content, and it is suggested that the vacancies may introduce deep trap levels and influence the intensity of YL band. This should be an important reason for the apparent change in the responsivity of the five detectors.

## 4. Conclusions

In summary, we have investigated the effect of Al content on the responsivity of AlGaN Schottky detectors by spectral response test and positron annihilation measurements. In our experiment, it was found that the higher the Al content, the lower responsivity of the AlGaN Schottky photodetectors. The positron annihilation measurements demonstrated that the concentration of Al or Ga vacancies was higher in the higher Al content Al_x_Ga_1-x_N detector samples (x<7%), which suggests that the Al or Ga vacancies play an important role in decreasing the responsivity of AlGaN Schottky detectors because the vacancy defects increase the recombination probability of photogenerated carriers.

## Figures and Tables

**Table 1:** Growth conditions and characterization parameters of the Al_x_Ga_1-x_N layer in T0–T4 samples.

Sample	TMGa Flux (umol/min)	TMAl Flux (umol/min)	Al Content (%)	FWHM of HRXRD (arc sec)		Carbon Impurity (atom/cm^3^)	Oxygen Impurity (atom/cm^3^)	Vacancy Concentration (/cm^3^)	Peak Responsivity (A/W)
				(002)	(102)				
T0	203	0	0	291	310	$5.6\times 10^{16}$	$6.22\times 10^{16}$	$2.57\times 10^{16}$	0.091
T1	81	2.00	2.60	298	306	$3.76\times 10^{16}$	$1.41\times 10^{17}$	$5.88\times 10^{16}$	0.076
T2	81	3.00	3.77	283	301	$3.54\times 10^{16}$	$1.45\times 10^{17}$	$1.07\times 10^{17}$	0.014
T3	81	4.50	5.44	284	304	$3.69\times 10^{16}$	$1.42\times 10^{17}$	$1.28\times 10^{17}$	0.012
T4	81	6.00	6.47	275	310	$4.01\times 10^{16}$	$1.52\times 10^{17}$	$2.55\times 10^{17}$	0.011

## Data Availability

The data that support the findings of this study are available from the corresponding author upon reasonable request.
